# Supplementary material for: Costs of hand hygiene for all in household settings: estimating the price tag for the 46 least developed countries
Source: BMJ Glob Health. 2021 Dec 16;6(12):e007361. doi: 10.1136/bmjgh-2021-007361 (PMC8679104; doi:10.1136/bmjgh-2021-007361)
Supplement: Supplementary data [file bmjgh-2021-007361supp001.pdf]

Online supplementary material

A. Additional background

- List of Least Developed Countries

B. Additional methodological information

- Model structure
- JMP imputation
- Formula for number of rural households with no hygiene service
- Electronic searches
- Intervention studies from which promotion price is derived
- Methods for estimating the cost of water

C. Input unit costs

D. Additional results

- Results for alternative intervention scenario excluding one-to-one promotion
- Scenarios for deterministic sensitivity analysis
- Deterministic sensitivity analysis results for promotion cost

Ross I, *et al. BMJ Global Health* 2021; 6:e007361. doi: 10.1136/bmjgh-2021-007361

A. Additional background

List of Least Developed Countries

**Americas and Caribbean (1)**		**Middle East and North Africa (3)**	
Haiti		Djibouti	Yemen
		Sudan	
**East Asia and the Pacific (7)**		**South Asia (4)**	
Cambodia	Solomon Islands	Afghanistan	Bhutan
Kiribati	Timor-Leste	Bangladesh	Nepal
Lao	Tuvalu		
Myanmar			
**Eastern and Southern Africa (15)**		**West and Central Africa (16)**	
Angola	Mozambique	Benin	Liberia
Burundi	Rwanda	Burkina Faso	Mali
Comoros	Somalia	Central African Republic	Mauritania
Eritrea	South Sudan	Chad	Niger
Ethiopia	Tanzania	Congo, D.R.	São Tomé and Principe
Lesotho	Uganda	The Gambia	Senegal
Madagascar	Zambia	Guinea	Sierra Leone
Malawi		Guinea-Bissau	Togo

Ross I, *et al. BMJ Global Health* 2021; 6:e007361. doi: 10.1136/bmjgh-2021-007361

B. Additional methodological information

Model structure

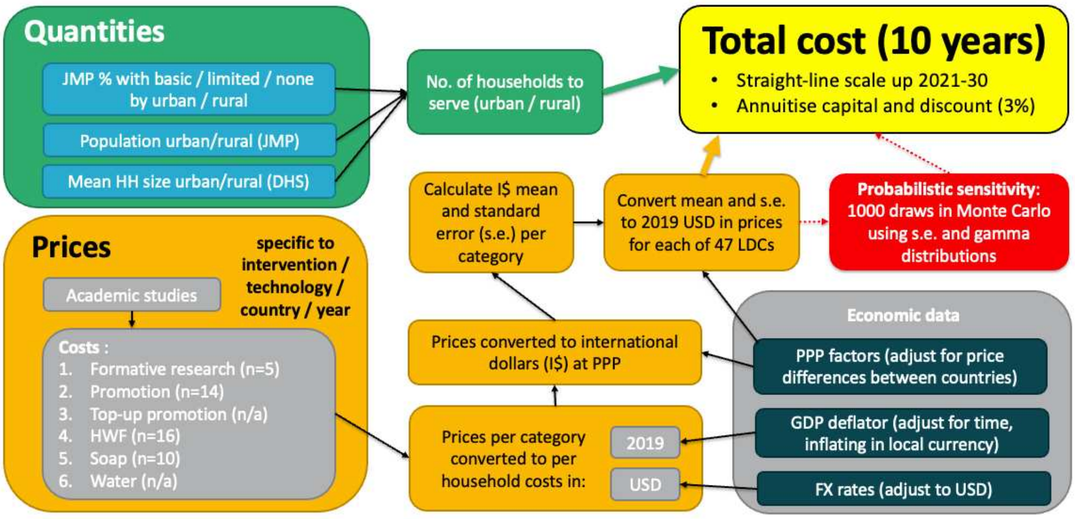

JMP imputation

The population of the five countries for which the LDC average is imputed represent only 6% of the total LDC population.

Prior coverage year applied instead of 2020, in cases of missing data

Country	No hygiene facility	Piped / non-piped water supply
Comoros	2016	2019
Djibouti	2020 LDC average* (21% urban, 30% rural)	2020
Eritrea	2020 LDC average* (21% urban, 30% rural)	2016
Liberia	2017	2020
Mauritania	2019	2020
Mozambique	2015	2020
Solomon Islands	2019	2020
South Sudan	2020 LDC average* (21% urban, 30% rural)	2020
Sudan	2020 LDC average* (21% urban, 30% rural)	2020
Tuvalu	2020 LDC average* (21% urban, 30% rural)	2018
Yemen	2017	2020

*LDC average here means the average proportion of people across all LDCs with no hygiene facility

Ross I, *et al. BMJ Global Health* 2021; 6:e007361. doi: 10.1136/bmjgh-2021-007361

Formula for number of rural households with no hygiene service

We used the below formula to calculate numbers of households to be served in rural areas, per country. An equivalent formula was used for urban areas.

$$A_r = \frac{B_r}{C_r} * D_r$$

where:

A_r is the number of rural households with "no hygiene service" in the country
B_r is the total rural population in the country (UN-DESA medium variant 2019)
C_r is the average rural household size in the country (latest DHS)
D_r is proportion of the rural population in the country with "no hygiene service" (JMP data)

Electronic searches

On 4th June 2021, we searched Google Scholar for records since 2015, just before the Hutton & Varughese (2016) study was finalised. Search terms were handwashing cost (without inverted commas), "soap expenditure" and "expenditure on soap". We reviewed the first 10 pages of results for each search, downloaded full texts, and word-searched them for "cost", "$", ”US", "price", and names/symbols of the currency of the study country.

Intervention studies from which promotion price is derived

Studies from which we extracted the price of hand hygiene promotion are listed below. Where source data excluded the costs of administration/management of the campaign, we attributed an uplift based on the average percentages for this cost from across studies that did so (24%).

Promotion interventions (12 studies reporting 14 interventions)

Borghi J, Guinness L, Ouedraogo J, Curtis V. Is hygiene promotion cost-effective? A case study in Burkina Faso. Trop Med Int Heal 2002; 7: 960–9.

Bikash Srot Kendra. Piloting hygiene promotion through routine immunisation in Nepal. 2017.

Freeman, M. C., Delea, M. G., Snyder, J. S., Garn, J. V., Belew, M., Caruso, B. A., … Gebremariam, A. (2021). The impact of a demand-side sanitation and hygiene promotion intervention on sustained behavior change and health in Amhara , Ethiopia : a cluster-randomized trial. MedRxiv. https://doi.org/10.1101/2021.07.15.21260587.

Briceño B, Chase C. Cost and Cost-Efficiency of Rural Sanitation and Handwashing Promotion: Activity-Based Costing and Experimental Evidence from Indonesia, India, Tanzania and Peru. 2014.

Pinfold J, Horan N. Measuring the effect of a hygiene behaviour intervention by indicators of behaviour and diarrhoeal disease. Trans R Soc Trop Med Hyg 1996; 90: 366–71.

Rajaraman D, Varadharajan KS, Greenland K, et al. Implementing effective hygiene promotion: Lessons from the process evaluation of an intervention to promote handwashing with soap in rural India. BMC Public Health 2014; 14. DOI:10.1186/1471-2458-14-1179.
Saadé C, Bateman M, Bendahmane DB. The Story of a Successful Public-Private Partnership in Central America. Handwashing for Diarrheal Disease Prevention. Arlington, Virginia: Basic Support for Child Survival Project (BASICS II), 2001.
Greenland K, Chipungu J, Curtis V, et al. Multiple behaviour change intervention for diarrhoea control in Lusaka, Zambia: a cluster randomised trial. Lancet Glob Heal 2016; 4: e966–77.
Evans B, Bates L, Halder A. Analysing the Value for Money of SHEWA-B in Bangladesh. 2015.
Waterkeyn J, Matimati R, Muringaniza A, et al. Comparative Assessment of Hygiene Behaviour Change and Cost-Effectiveness of Community Health Clubs in Rwanda and Zimbabwe. Healthc Access - Reg Overviews 2019. DOI:10.5772/intechopen.89995.
Biran A, White S, Awe B, et al. A cluster-randomised trial to evaluate an intervention to promote handwashing in rural Nigeria. Int J Environ Health Res 2020; 00: 1–16.
George CM, Monira S, Sack DA, et al. Randomized controlled trial of hospital-based hygiene and water treatment intervention (CHoBI7) to reduce cholera. Emerg Infect Dis 2016; 22: 233–41.

Methods for estimating the cost of water

Separately for urban and rural, we estimated a per country average price of water based on: (i) the proportion of households using piped improved water supply (JMP data for 2020);[1] (ii) the average national tariff per m^3 reported by the International Benchmarking Network for Water and Sanitation Utilities.[2] Households using piped improved were assumed to pay the IBNET tariff. Unconnected households were assumed to pay double that tariff, an approximation in the absence of data, to reflect their likely increased economic cost of water due to travel time, and/or the cost of maintaining self-supplied water assets. We combined the above prices with an assumed volume per person per day of 1.5 litres, to estimate an annual cost of water for handwashing. The volume estimate is based on: (i) an average of measured volume data reported by Whinnery et al.[3] for three types of barrel and tap technologies, tippy tap, and jug/basin; (ii) the assumptions that, in real life, people wash their hands for 10 seconds an average of four times per day.

C. Input unit costs

Promotion activities – cost per household

Author / date	Country	Technology / approach	As reported in study			Cost / HH (2019)		DOI / URL
			Value	Curr-ency	Data year	US dollars	Int'l dollars	
Borghi 2002	Burkina Faso	Monthly house-to-house visits, discussions at health centres, weekly theatre performances, comic radio broadcasts, training teachers	7.1	USD	1999	11.2	32.5	https://doi.org/10.1046/j.1365-3156.2002.00954.x
Rajamaran 2014	India	Community and school-based events (film, skits, pledging)	647	INR	2011	12.8	42.5	https://doi.org/10.1186/1471-2458-14-1179
Biran 2020	Nigeria	Community-wide meeting, followed by short compound level discussions.	8,626	NGN	2015	41.6	94.5	https://doi.org/10.1080/09603123.2020.1788712
Evans 2015	Bangladesh	Community-based promoters delivered messages using equipment such as flash cards, plus a mass media campaign (radio spots, video spots)	0.68*	USD	2007	5.1	13.8	vfmwash990848521.wordpress.com
Waterkeyn 2020	Zimbabwe	24 group sessions delivered over six months with homework, individual follow-up with almost universal participation	29.2	USD	2012	33.9	68.6	http://dx.doi.org/10.5772/intechopen.89995
Waterkeyn 2020	Rwanda	24 group sessions delivered over six months with homework, individual follow-up with almost universal participation	76.5	USD	2013	67.0	189.9	http://dx.doi.org/10.5772/intechopen.89995
Greenland 2016	Zambia	Women's forums delivered in neighbourhoods; roadshows delivered in public gathering spaces; clinic-based 'circle of mothers' sessions with monthly prize draws; call-in programmes on local radio	198	ZMK	2013	25.0	69.3	https://doi.org/10.1016/S2214-109X(16)30262-5

George 2016	Bangladesh	Hospital-based promotion to cholera patient and family	29.76	USD	2013	38.5	103.6	https://doi.org/10.3201/eid2202.151175
Freeman 2021	Ethiopia	Community mobilization and commitment events, community conversations, household counseling visits with caregivers	1,215	ETB	2017	53.0	148.8	https://doi.org/10.1101/2021.07.15.21260587
Pinfold 1996	Thailand	Handwashing and dishwashing campaign using music, theatre, posters over 3 months	4.2	GBP	1991	4.5	11.0	https://doi.org/10.1016/S0035-9203(96)90507-6
Saade 2001	Guatemala	Mass media campaign plus minor school activities	1.4	USD	1997	2.8	5.4	https://www.ircwash.org/sites/default/files/Saade-2001-Story.pdf
Briceno 2014	Tanzania	National mass media campaign, promotional events, personal contacts at the ward level	3.3*	USD	2010	18.7	46.0	unpublished - reference above
Briceno 2014	Peru	Mass media, public marketing events, activities in communities and schools (training of teachers / medics / community leaders, HWWS demos)	3.6*	USD	2010	19.4	37.2	unpublished - reference above
Bikash Srot Kendra 2021	Nepal	5 x hygiene sessions alongside child's immunisation	16.5	USD	2015	19.6	65.2	unpublished - reference above

* denotes per person value reported in study, converted to per household for 2019 columns

Handwashing facilities – cost per household

Author / date	Country	Technology / approach	As reported in study			Cost / HH (2019)		DOI / URL
			Value	Curr-ency	Data year	US dollars	Int'l dollars	
**HWF (purpose-built)**								
Hutton 2016	Indonesia	Standing basin with tap (as reported to Hutton)	150,000	IDR	2014	12.4	37.1	http://hdl.handle.net/10986/23681
Hutton 2016	Uganda	Standing basin with tap (as reported to Hutton)	95,000	UGX	2014	31.5	89.9	http://hdl.handle.net/10986/23681
Hutton 2016	Uganda	Standing basin without tap (as reported to Hutton)	70,000	UGX	2014	23.2	66.2	http://hdl.handle.net/10986/23681
Luby 2018	Bangladesh	Plastic bucket on plastic stand w/ plastic bowl and soapy water bottle.	445	BDT	2012	7.9	21.3	https://doi.org/10.1016/S2214-109X(17)30490-4
Daily Star	Bangladesh	Happy tap (plastic integrated handwashing unit)	1,550	BDT	2020	18.4	49.3	https://bit.ly/3klgR0e
Whinnery 2016	Kenya	Povu poa - economical foaming soap dispenser and a hygienic, water-efficient tap	12	USD	2016	14.1	35.1	https://doi.org/10.9745/GHSP-D-16-00022
George 2016	Bangladesh	Plastic bucket on plastic stand w/ plastic bowl and soapy water bottle	12	USD	2013	15.5	41.8	https://doi.org/10.3201/eid2202.151175
Freedman 2017	Kenya	60-liter plastic bucket with a tight-fitting lid and tap, metal stand, and plastic washbasin	1,500	KSH	2011	25.1	62.4	https://doi.org/10.2166/washdev.2017.010
Rajasingham 2018	Kenya	Improved storage containers with a narrow mouth, lid, and spigot on a metal stand	15	USD	2005	36.8	91.6	https://doi.org/10.2166/wh.2018.149
Mmanga 2020	Malawi	Handwashing station using a 20 or 50 L bucket and tap system	20	USD	2017	22.6	60.7	https://doi.org/10.1016/j.pce.2020.102862
Hutton 2016	Bangladesh	Stool with 10 litre bowl (as reported to Hutton)	293	BDT	2014	4.6	12.4	http://hdl.handle.net/10986/23681

Hutton 2016	Kenya	20 liters plastic drum with tap (as reported to Hutton)	450	KGX	2014	6.1	15.1	http://hdl.handle.net/10986/23681
Hutton 2016	Bangladesh	Kitchen sink (as reported to Hutton)	800	BDT	2014	12.5	33.7	http://hdl.handle.net/10986/23681
van de Reep 2010	Mozambique	Washbasin	982	MZN	2008	27.0	71.7	https://www.ircwash.org/sites/default/files/Reep-2010-Cost.pdf
Hutton 2016	Kenya	20 liters plastic drum with tap (as reported to Hutton)	450	KGX	2014	6.1	15.1	http://hdl.handle.net/10986/23681
Hutton 2016	Mali	Jug and bowl with plughole (as reported to Hutton)	3,000	XOF	2014	5.6	15.5	http://hdl.handle.net/10986/23681
**HWF (home-made)**								
Borghi 2002	Burkina Faso	Jug for pouring water	0.8	USD	1999	1.3	3.6	https://doi.org/10.1046/j.1365-3156.2002.00954.x
van de Reep 2010	Mozambique	Clay pot and cup	26	MZN	2008	0.7	1.9	https://www.ircwash.org/sites/default/files/Reep-2010-Cost.pdf
van de Reep 2010	Mozambique	Plastic bucket and cup	51	MZN	2008	1.4	3.7	https://www.ircwash.org/sites/default/files/Reep-2010-Cost.pdf
Hutton 2016	Uganda	Tippy Tap (as reported to Hutton)	3,500	UGX	2014	1.2	3.3	http://hdl.handle.net/10986/23681

Annual soap expenditure – cost per household

Author / date	Country	Technology / approach	As reported in study			Cost / HH (2019)		DOI / URL
			Value	Curr-ency	Data year	US dollars	Int'l dollars	
Borghi 2002	Burkina Faso	Expenditure on balls of soap from market (authors assume 10% for handwashing)	3.6	USD	1999	5.7	16.6	https://doi.org/10.1046/j.1365-3156.2002.00954.x
van de Reep 2010	Mozambique	Expenditure on soap (we assume 30% for handwashing)	243	MZN	2008	6.7	17.8	https://www.ircwash.org/sites/default/files/Reep-2010-Cost.pdf
George 2016	Bangladesh	Expenditure on detergent for soapy water (implies 100% for handwashing)	15.6	USD	2013	20.2	54.3	https://doi.org/10.3201/eid2202.151175
Woode 2018	Ghana	Expenditure on soap (we assume 30% for handwashing)	53.4	GHS	2013	21.7	55.8	https://doi.org/10.1016/j.heliyon.2018.e00841
Briceno 2014	Tanzania	Expenditure on soap (implies 100% for handwashing)	34.2	USD	2010	39.5	97.4	unpublished - reference above
Strukova 2007	Honduras	Expenditure on soap -and water (cannot be disaggregated)	171	LCU	2005	14.2	33.0	https://bit.ly/3qk4S6V
Abramov-sky 2019	Philippines	Expenditure on soap (we assume 30% for handwashing)	742	PHP	2015	15.5	41.4	https://doi.org/10.1920/wp.ifs.2019.1519
Briceno 2014	Peru	Expenditure on soap (implies 100% for handwashing)	23.1	USD	2010	24.8	47.7	unpublished - reference above
Hussam 2016	India	Expenditure on soap (implies 100% for handwashing)	720	INR	2015	11.8	39.0	http://cega.berkeley.edu/assets/cega_events/114/Hussam_Handwashing_paper.pdf
Hutton 2016	Kenya	Expenditure on multi-purpose bar soap (assume 100% for handwashing)	576	KGX	2014	7.7	19.3	http://hdl.handle.net/10986/23681

D. Additional results

Results for alternative intervention scenario excluding one-to-one promotion

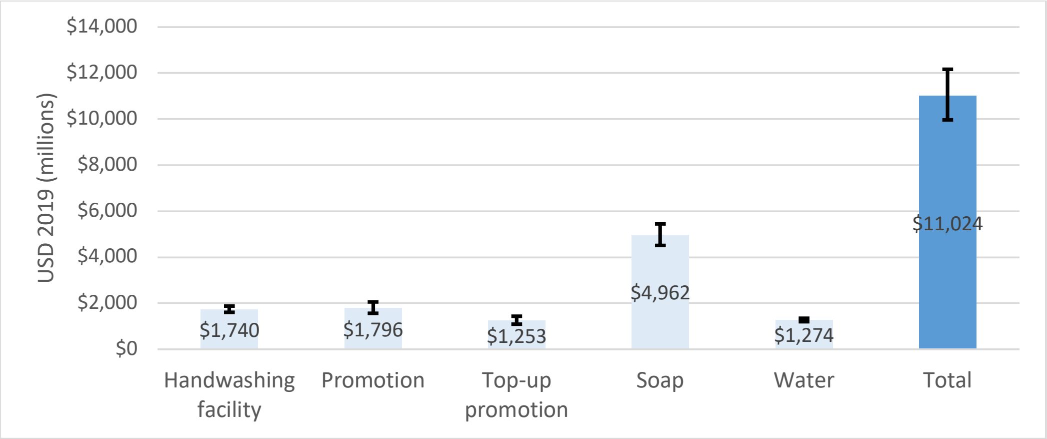

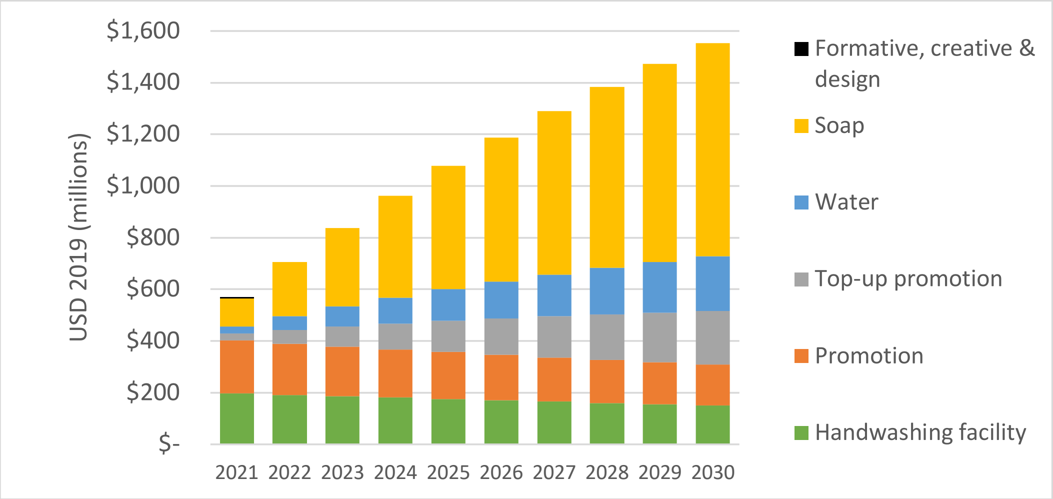

Scenarios for deterministic sensitivity analysis

		Variables		
		**Lower cost**	**Base case**	**Higher cost**
**Promotion**	Promotion price	lower bound of 95% CI (I$ 38)	mean (I$ 86)	upper bound of 95% CI (I$ 134)
	Top-up promotion price	20% every 2 years	25% every 1 year	35% every 1 year
	Useful life of promotion	7 years	5 years	3 years
**HWF**	HWF price	lower bound of 95% CI (I$ 32)	mean (I$ 45)	upper bound of 95% CI (I$ 58)
	HWF useful life	7 years	5 years	3 years
	Home-made HWF instead of purpose built	“tippy-tap” or repurposed jug/bowl, with a useful life of 2 years (mean prices of n=4 studies)	purpose-built	n/a
**Other**	Annual soap expenditure	lower bound of 95% CI (I$ 29)	mean (I$ 46)	upper bound of 95% CI (I$ 63)
	Water volume used for handwashing (litres / person / day)	1	1.5	2
	Discount rate (based on IDSI/GHCC reference cases – see main body)	7%	3%	0.1%
	Economies of scale	Prices of promotion, HWFs and soap in year 2 are 10% lower than year 1, further 8% lower in year 3, etc. such that price from year 7 onwards is 30% lower than year 1 & remains constant.	no change	n/a

Deterministic sensitivity analysis results for promotion cost

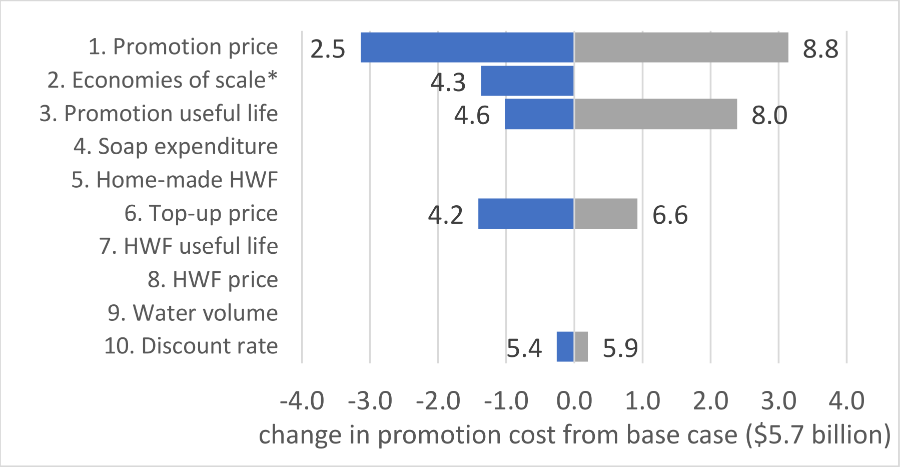

References for supplementary materials

1 UNICEF & WHO. Progress on household drinking water, sanitation and hygiene 2000-2017. Special focus on inequalities. New York, 2019.

2 IBNET. IBNET tariffs database. Int. Benchmarking Netw. Water Sanit. Util. 2021. https://tariffs.ib-net.org/ (accessed Feb 15, 2021).

3 Whinnery J, Penakalapati G, Steinacher R, Wilson N, Null C, Pickering AJ. Handwashing with a water-efficient tap and low-cost foaming soap: the Povu Poa 'Cool Foam' system in Kenya. *Glob Heal Sci Pract* 2016; **4**: 336–41.
